# Estimated glomerular filtration rate and arterial stiffness in Japanese population: a secondary analysis based on a cross-sectional study

**DOI:** 10.1186/s12944-019-0997-4

**Published:** 2019-03-04

**Authors:** Yun-Fen Chen, Chi Chen

**Affiliations:** 1 Department of rheumatism for nephropathy, Guizhou Provincial People’s Hospital, 83# Zhongshaneast Road, Guiyang, 550001 Guizhou China; 2 Department of Immunology and Microbiology, Guiyang College of Traditional Chinese Medicine, 84# ShiDong Road, Guiyang, 550001 Guizhou China

**Keywords:** Estimated glomerular filtration rate, Brachial-ankle pulse wave velocity, Nonlinearity

## Abstract

**Background:**

Evidence regarding the relationship between estimated glomerular filtration rate (eGFR) and arterial stiffness is limited, and the data analysis is not sufficient to clarify the true relationship between the two. We aimed to investigate the relationship between eGFR and brachial-ankle pulse wave velocity (baPWV) in Japanese.

**Methods:**

The present study was a cross-sectional study. Nine hundred twelve Japanese men and women, aging 24—84 years old, received a health medical check-up program including the results from baPWV inspection and various standardized questionnaires in a health examination center in Japan. The main outcome measures included eGFR, baPWV, fatty liver and postmenopausal status. Abdominal ultrasonography was used to diagnose fatty liver. Postmenopausal state was defined as beginning 1 year after the cessation of menses.

**Results:**

The average age of the 912 selected participants was 51.5 ± 9.6 years old, and about 57.6% of them were male. The participants’ eGFR distribution was median 69.29 (min 39, max 122.28). The results of multivariate linear regression showed eGFR was not independently associated with baPWV after adjusting potential confounders (β = − 1.11, 95%CI -2.25 to 0.03), this is inconsistent with the result of eGFR (quartile) as a categorical variable (p for trend was 0.038). A non-linear relationship was detected between eGFR and baPWV, whose point was 77.05. The effect sizes and the confidence intervals of the left and right sides of inflection point were − 2.80 (− 4.41 to − 1.19) and 1.84 (− 0.50, 4.17), respectively. Subgroup analysis showed, the change in the elderly population is more pronounced (P for interaction = 0.018; − 2.83 with ≤60 year vs − 6.12 with > 60 year). The same trend was also seen in hypertensive people (P for interaction = 0.018; − 4.55 with hypertension vs − 0.82 with non-hypertension).

**Conclusion:**

The relationship between eGFR and baPWV is non-linear. eGFR was negatively related to baPWV when eGFR is less than 77.05.

## Introduction

Although carotid-femoral pulse wave velocity (cfPWV) is served as a gold standard technique to measure arterial stiffness, the technical precision required for carotid pulse acquisition and the intimate nature of femoral pulse acquisition hinders the widespread use of this methodology in the clinic [1]. Therefore, brachial-ankle pulse wave velocity (baPWV) is used in routine clinical settings in Japan due to its ease of use [2]. To date, an increasing number of publications on baPWV methodology have come from western countries since 2009 [3]. Large-scale clinical studies in the United States like Atherosclerosis Risk in Communities (ARIC) study and the Bogalusa Heart Study have used baPWV as an indicator for assessing arterial stiffness [4, 5].

Glomerular filtration rate (GFR) was used to describe the flow rate of filtered fluid through the kidney. Compared with GFR, estimated glomerular filtration rate (eGFR), a simpler and more applicable surrogate marker to general population studies, has been widely used clinically to diagnose chronic kidney disease (CKD) and to assess renal function [6]. Prior studies have demonstrated that increased arterial stiffness plays a key role in the progression of CKD [7–11]. However, most of these studies did not consider nonlinearity in the data analysis process and did not perform subgroup analysis.

In this study, our primary objective was to investigate the relationship between eGFR and baPWV. We performed a secondary data analysis based on an existing data that comes from a previously published paper [12]. In the original paper, the author has investigated the correlation between γ-glutamyl transpeptidase and baPWV. While in secondary analysis, eGFR was used as independent variable, and outcome variable and other covariates are consistent with those in the original.

## Methods

### Data source

We freely downloaded the raw data uploaded by Fukuda, et al. from the “DATADRYAD” database (www.datadryad.org). Since Fukuda, et al. have authorized the ownership of the original data to the datadryad website, we can use this data to perform secondary data analysis based on different scientific assumptions. (Dryad data package: Fukuda T, Hamaguchi M, Kojima T, Ohshima Y, Ohbora A, Kato T, Nakamura N, Fukui M (2014) Data from: Association between serum γ-glutamyltranspeptidase and atherosclerosis: a population-based cross-sectional study. Dryad Digital Repository. 10.5061/dryad.m484p).

Variables included in the database file were as follows: age, diastolic blood pressure (DBP), body mass index (BMI), alanine aminotransferase (ALT), systolic blood pressure (SBP), aspartate transaminase (AST), γ-glutamyltranspeptidase (GGT), fasting glucose, uric acid, total cholesterol (TC), low density lipoprotein (LDL), baPWV, estimated glomerular filtration rate (eGFR), sex, smoking status, exercise, fatty liver disease, menopausal status, high-density lipoprotein cholesterol (HDL-C), alcohol consumption, ankle-brachial index (ABI) and triglyceride (TG).

### Study population

It is important to note that Fukuda and his collaborators completed the entire study. In order to give the readers a clear understanding of the design and implementation steps of the entire study, we have a brief retelling of this. Fukuda and his partners conducted a cross-sectional study at Medical Health Checkup Center of Murakami Memorial Hospital, Gifu city, Japan from March 2004 to December 2012. The participants involved in the study received a medical health check-up programme including pulse wave velocity and abdominal ultrasonography. A total of 1, 445 participants were recruited and selected according to exclusion standard. Exclusion standards: (1) participants received hormone replacement therapy, (2) participants took oral contraceptives, (3) Hepatitis B virus antigen and hepatitis C virus antigen was positive; (4) The participants were pregnant, (5) ankle-brachial index (ABI) was less than 0.95. Since the study was based on a secondary analysis of past data, and the patients’ personal information in the original data is anonymous, there is no need to get informed consent from the participants. The ethical license has been elaborated in the published paper.

### Measurement of baPWV, eGFR and other covariants

Fukuda et al [12] completed the entire study. In order to allow understanding of the entire research process more clearly, we have outlined the steps of the study here.

baPWV and ABI were measured using an automatic waveform analyzer (Colin Medical Technology, Komaki, Japan). The participants took the supine position and rested in a quiet and suitable temperature room for 5 min, and then ECG electrodes and heart sound microphone were placed on both wrists and the left edge of the sternal border respectively. Cuffs connected to a plethysmographic sensor and an oscillometric pressure sensor were wrapped on the branchia and ankles. Takuya Fukuda et al. then calculated the path lengths from the suprasternal notch to the brachium (Lb) and from the suprasternal notch to the ankle (La), and then automatically obtained the delay time of the ascending point of the brachial waveform to the ascending point of each ankle waveform (DTba). Finally, they calculated baPWV by formula (La-Lb) / DTba. The intraobserver and interobserver coefficients of variation were reported to be 10% (r = 0.87, *p* < 0.01) and 8.4% (r = 0.98, p < 0.01), respectively.

Fukuda, et al. calculated eGFR according to the Japanese Society of Nephrology model. The model is as follows: eGFR = 194 × Cr − 1.094 × age− 0.287 (mL/min/1.73 m^2^) for men, and eGFR was multiplied by a correction factor of 0.739 for women.

Fukuda et al. diagnosed fatty liver by abdominal ultrasongraphy (Aloka SSD-650CL (Aloka Co, Ltd., Tokyo, Japan)).One gastroenterologist diagnosed fatty liver by ultrasonographic images stored in a computer without reference to other individual data of any of the participants. Of the four known criteria (hepatorenal echo contrast, liver brightness, deep attenuation and vascular blurring), the participants were required to have hepatorenal contrast and liver brightness to be given a diagnosis of fatty liver.

Fukuda et al. used a standardized questionnaire with all participants by the same trained team of interviewers. They evaluated alcohol consumption by asking the participants about (1) the amount and type of alcoholic beverages consumed per week; (2) the total amount of alcohol consumed per week (grams). Alcohol consumption was then categorized into four grades with < 40 g/week, 40–140 g/week, 140–280 g/week and > 280 g/week. Smoking status was divided into two groups of non-smoker or ex-smoker, and current smoker. For evaluating sports or recreational activities, interviewers asked participants the type, duration and frequency of sports or recreational activities. Any kind of sport regularly at least once a week was defined as regular exercisers. The postmenopausal state was defined as beginning 1 year after the cessation of menses.

### Statistical analysis

The first step in data analysis is to present the distribution of baseline data of patients included in this study in different eGFR groups (Quartile). We expressed continuous variables as mean ± standard deviation (normal distribution) or median (quartile) (skewed distribution). We expressed categorical variables in frequency or as a percentage. We used χ2 (categorical variables), One-Way ANOVA (normal distribution), or Kruskal-Wallis H test (skewed distribution) to calculate for differences among different eGFR groups.

The second step of data analysis could be summarized as: (1) is there any relationship between eGFR and baPW, is it linear or nonlinear? (2) What factors interfered with or modified the relationship between them? (3) What was the independent effect on eGFR and baPWV when we expel the effects of these potential confounders or modifiers? According to the above analysis principle, we used univariate and multivariate linear regression model to evaluate associations with eGFR and baPWV. According to the recommendation of STROBE statement [13], we constructed three models including an unadjusted model, a model adjusted to demographics and a fully-adjusted model. For the fully-adjusted model, the adjusted variables are the relevant covariates that may affect baPWV and (or) eGFR as reported in previous studies [14–20]. In addition, the subgroup analyses were performed using stratified linear regression models. Tests for effect modification by subgroup used interaction terms between subgroup indicators, followed by the likelihood ration test.

To ensure the robustness of the data analysis, we performed the following sensitivity analysis:

1. We converted the eGFR into a categorical variable by quartile. The purpose was to verify the results of eGFR as a continuous variable and to observe the possibility of nonlinearity.
2. Linear regression is a linear model, so the relationships between the independent variables and the dependent variable were linear. However, in biomedical data analysis, the relationship between the dependent variables and the independent variable are often nonlinear. Therefore, we used a generalized additive model to deal with nonlinear relationships.
3. If the relationship between eGFR and baPWV is nonlinear, a two-piecewise linear regression model would be performed to calculate the threshold effect of the eGFR on baPWV according to the smoothing plot. The saturation level of eGFR at which the relationship between baPWV and eGFR level began to change and became notable was determined using a recursion algorithm. The inflection point was moved along a pre-defined interval and detected the inflection point that gave the maximum model likelihood. We determined the best fit model based on the *P* value of log likelihood ratio tests. If P value is greater than 0.05, it is considered that there is no difference between the linear fitting model (linear regression model) and the nonlinear fitting model (two-piecewise linear regression model), and we can use linear model to fit the relationship between eGFR and baPWV. Conversely, if the *P*-value of the log-likelihood ratio test is less than 0.05, it is considered that the linear fitting model is significantly different from the non-linear fitting model, and the nonlinear fitting model is needed to clarify the relationship between eGFR and baPWV.

All the analyses were performed with the statistical software packages R (http://www.r-project.org, The R Foundation) and EmpowerStats (http://www.empowerstats.com, X&Y Solutions, Inc., Boston, MA). *P* values less than 0.05 (two-sided) were considered statistically significant.

## Results

### The selection of participants

Of the 1445 participants, 533 participants were excluded from this study. Of the 533 excluded participants, 433 received medications, 1 took oral contraceptive, 66 received hormone replacement therapy, 26 whose hepatitis B and hepatitis C antigen was positive, 1 was in gestational age, and 6 had ABI less than 0.96, leaving 912 selected participants for data analysis.

### Baseline characteristics of participants

Baseline characteristics of selected participants according to quartiles of eGFR are shown in Table 1. In general, the average age of the 912 selected participants was 51.5 ± 9.6 years old, and about 57.6% of them were male. The participants’ eGFR distribution was median 69.289 (min 39, max 122.28). No statistically significant differences were detected in AST, ALT, ABI, smoking status, exercise status, fatty liver among different eGFR groups (all *p* values > 0.05). Participants with the highest eGFR (Q4) were younger, consisted of more females and were postmenopausal, and had a lower BMI, SBp, DBp, Log_2_GGT, FBS, uric acid, TC, TG, LDL-C, baPWV and alcohol consumption than those with the lower eGFR (Q1-Q3). The opposite pattern was observed in HDL-C.

**Table 1:** Baseline characteristics of participants

eGFR (quartile)	Q1 (39.00–61.82)	Q2 (62.00–69.00)	Q3 (69.29–78.00)	Q4 (78.00–122.28)	*P*-value	*P*-value*
N (cases)	225	224	235	228		
Age mean (std), year	55.42 (8.58)	53.19 (8.04)	49.79 (9.94)	46.26 (9.05)	< 0.001	< 0.001
BMI mean (std), kg/m^2^	23.54 (2.79)	23.15 (2.83)	23.04 (3.01)	22.79 (3.74)	0.028	0.008
SBp mean (std), mmHg	122.60 (13.95)	120.73 (14.83)	121.00 (15.19)	116.67 (15.28)	< 0.001	< 0.001
DBp mean (std), mmHg	78.15 (9.61)	76.24 (9.97)	76.64 (10.34)	73.55 (9.62)	< 0.001	< 0.001
AST mean (std), U/L	21.45 (7.61)	21.05 (8.00)	20.86 (7.89)	20.06 (8.79)	0.316	0.115
ALT mean (std), U/L	23.33 (13.17)	22.48 (14.19)	22.89 (13.70)	22.04 (16.02)	0.799	0.374
Log_2_GGT mean (std) IU/L	4.47 (0.87)	4.34 (0.87)	4.39 (0.78)	4.23 (0.85)	0.026	0.003
FBS mean (std), mg/dl	101.70 (20.82)	97.91 (11.90)	97.07 (10.12)	95.60 (10.05)	< 0.001	< 0.001
Uric acid mean (std), mg/dl	5.85 (1.46)	5.21 (1.23)	5.24 (1.21)	4.72 (1.36)	< 0.001	< 0.001
TC mean (std), mg/dl	214.29 (35.46)	213.60 (33.37)	208.66 (37.60)	202.87 (36.30)	0.002	0.003
Triglycerides mean (std), mg/dL	113.17 (72.46)	96.12 (58.85)	102.71 (95.93)	87.47 (64.11)	0.003	< 0.001
HDL-C mean (std), mg/dL	51.43 (14.25)	53.32 (14.63)	53.67 (14.72)	55.68 (14.56)	0.021	0.009
LDL-C mean (std), mg/dL	132.15 (31.87)	131.84 (29.45)	126.26 (32.87)	122.16 (31.50)	0.001	0.002
ABI	1.20 (0.12)	1.25 (0.08)	1.20 (0.09)	1.18 (0.07)	0.338	0.095
baPWV mean (std), cm/s	1510.68 (307.37)	1442.92 (252.29)	1392.46 (198.00)	1319.37 (166.24)	< 0.001	< 0.001
Sex					< 0.001	–
male n(%)	164 (72.89%)	134 (59.82%)	168 (71.49%)	126 (55.26%)		
female n(%)	61 (27.11%)	90 (40.18%)	67 (28.51%)	102 (44.74%)		
Smoking status					0.132	–
None n(%)	185 (82.22%)	181 (80.80%)	180 (76.60%)	169 (74.12%)		
Current n(%)	40 (17.78%)	43 (19.20%)	55 (23.40%)	59 (25.88%)		
Ex-Smoker n%)					0.333	–
no n(%)	105 (46.67%)	121 (54.02%)	114 (48.51%)	121 (53.07%)		
Yes n(%)	120 (53.33%)	103 (45.98%)	121 (51.49%)	107 (46.93%)		
Alcohol consumption					0.007	–
0–40 g/week n(%)	142 (64.25%)	153 (68.61%)	125 (54.11%)	161 (71.88%)		
40–140 g/week n(%)	32 (14.48%)	34 (15.25%)	57 (24.68%)	27 (12.05%)		
140–280 g/week n(%)	24 (10.86%)	21 (9.42%)	25 (10.82%)	18 (8.04%)		
> 280 g/week n(%)	23 (10.41%)	15 (6.73%)	24 (10.39%)	18 (8.04%)		
Regular exerciser					0.277	–
no n(%)	178 (80.54%)	172 (78.54%)	180 (77.59%)	189 (84.38%)		
yes n(%)	43 (19.46%)	47 (21.46%)	52 (22.41%)	35 (15.62%)		
Fatty liver					0.138	–
None n(%)	145 (64.73%)	163 (72.77%)	172 (73.19%)	166 (72.81%)		
Moderate or severe n(%)	79 (35.27%)	61 (27.23%)	63 (26.81%)	62 (27.19%)		
Menopausal status					< 0.001	–
Postmenopausal n(%)	23 (37.70%)	19 (21.11%)	30 (44.78%)	66 (64.71%)		
no n(%)	38 (62.30%)	71 (78.89%)	37 (55.22%)	36 (35.29%)		

Noted: P* was calculated by Kruskal-Wallis H test

BMI, body mass inex; ALT, alanine aminotransferase; AST, aspartate aminotransferase;

FBS, fasting blood glucose; TC, total cholesterol; HDL-C, ligh density lipoprotein;

LDL-C, low-density lipoprotein; ABI, ankle brachial index; baPWV, brachial-ankle pulse wave velocity

### Univariate and multivariate analysis

The results of univariate and multivariate linear regression model are shown in Table 2. The non-adjusted model showed that for each additional unit of eGFR, the baPWV is reduced by 6.39 (95% CI: -7.65, − 5.12). We also showed the minimally-adjusted model and the fully-adjusted model. Compared with the unadjusted model (β = − 6.39 (− 7.65, − 5.12), eGFR in the minimally-adjusted model (only adjusted for sex and age) was still negatively correlated with baPWV, but the effect size was significantly reduced (β = − 2.11 (− 3.36, − 0.85). In the fully-adjusted model, the effect size of was − 1.11 (− 2.25, 0.03) after adjusting for age, sex, BMI, SBp, DBp, AST, ALT, log_2_GGT, fasting glucose, serum uric acid, TC, TG, LDL-C, HDL-C, smoking status, ex-smoker, exercise status, alcohol consumption, ABI and fatty liver.

**Table 2:** The results of univariate and multivariate analyses

	non-adjusted model β(95%CI)	Minimally-adjusted model β(95%CI)	Fully-adjusted model β(95%CI)
eGFR	−6.39 (−7.65, − 5.12)	− 2.11 (− 3.36, − 0.85)	− 1.11 (− 2.25, 0.03)
eGFR (quartile)			
Q1	Ref	Ref	Ref
Q2	−67.76 (− 111.52, − 24.00)	− 31.36 (− 70.44, 7.72)	−17.52 (−50.80, 15.76)
Q3	− 118.22 (− 161.46, − 74.97)	−47.61 (− 86.83, − 8.39)	− 34.62 (− 68.65, − 0.59)
Q4	− 191.31 (− 234.87, − 147.74)	− 65.99 (− 107.57, − 24.41)	−35.35 (− 72.34, 1.64)
p for trend	0.0001	0.0001	0.038

CI: Confidence interval

Non-adjusted model: we did not adjust any covariate

Minimally-adjusted model: we only adjusted age and sex

fully-adjusted model: we adjusted age, sex, BMI, SBp, DBp, AST, ALT, log2GGT, fasting glucose, serum uric acid, TC, TG, LDL-C, HDL-C, smoking status, ex-smoker, exercise status, alcohol consumption, ABI and fatty liver

For the purpose of sensitivity analysis, we converted the eGFR into categorical variable by quartile and calculated P for trend (Table 2). In the fully adjusted model, compared with the reference Q1 group, the estimated decrease of baPWV in the Q2, Q3 and Q4 group were 17.52, 34.62 and 35.35, respectively. The P for trend was 0.038. The results were not consistent with the results of eGFR as a continuous variable. This kind of non-equidistant changes in effect size suggested that there may be a nonlinear relationship between eGFR and baPWV.

### The analyses of non-linear relationship

**Fig. 1:**
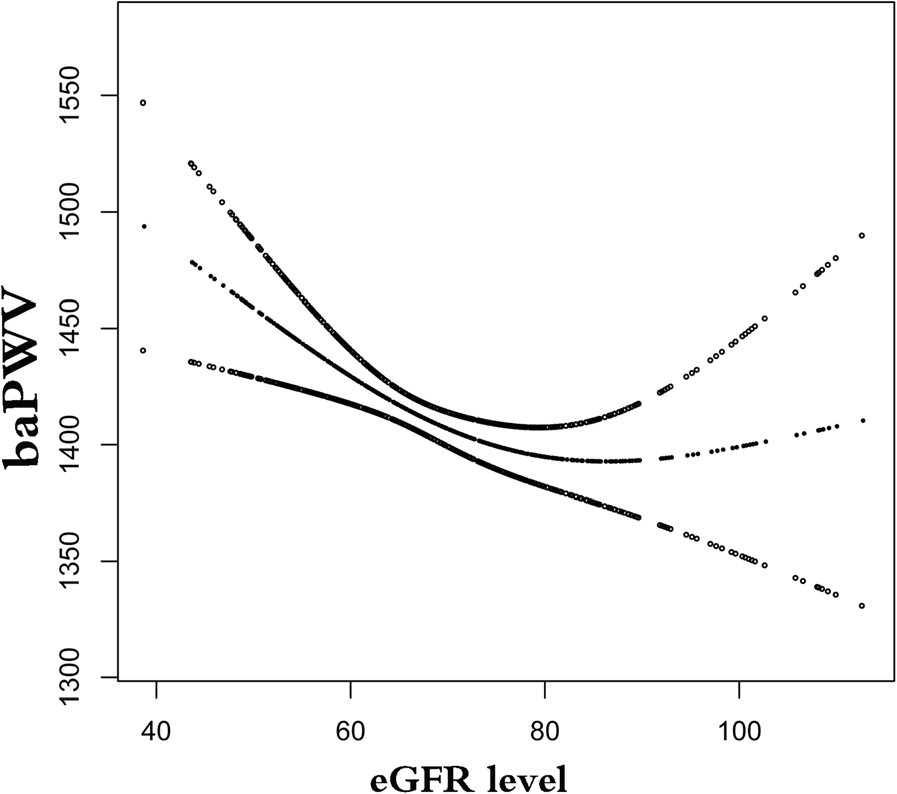
The relationship between eGFR and baPWV. A nonlinear relationship between them was detected after adjusting for age, sex, BMI, SBp, DBp, AST, ALT, log_2_GGT, fasting glucose, serum uric acid, TC, TG, LDL-C, HDL-C, smoking status, ex-smoker, exercise status, alcohol consumption, ABI and fatty liver

In this present study, we analyzed the non-linear relationship between eGFR and baPWV (Fig. 1). The result of smooth curve through the generalized additive model showed that the relationship between eGFR and baPWV was non-linear (age, sex, BMI, SBp, DBp, AST, ALT, log_2_GGT, fasting glucose, serum uric acid, TC, TG, LDL-C, HDL-C, smoking status, ex-smoker, exercise status, alcohol consumption, ABI and fatty liver). We compared linear regression model (fitting the relationship between eGFR and baPWV by a linear) and two-piecewise linear regression model (fitting the relationship between eGFR and baPWV by a curve) (Table 3). The P for log likelihood ratio test is less than 0.05. This result indicates that the two-piecewise linear regression model should be used to fit the relationship between X and Y. By two-piecewise linear regression model and recursive algorithm, we calculated the inflection point was 77.05. On the left of inflection point, the effect size, 95%CI and *P* value were − 2.80, − 4.41 to − 1.19 and 0.007, respectively. However, on the right side of the inflection point, we did not observe an association between eGFR and baPWV (1.84 (− 0.50, 4.17), *P* = 0.123).

**Table 3:** The results of two-piecewise linear model

	baPWV (β, 95%CI)
Fitting model by standard linear regression	−1.11 (− 2.25, 0.03)
Fitting model by two-piecewise linear regression	
Inflection point of eGFR	77.05
≤77.05	− 2.80 (− 4.41, − 1.19)
> 77.05	1.84 (− 0.50, 4.17)
P for log likelihood ratio test	0.004

CI: Confidence interval

we adjusted age, sex, BMI, SBp, DBp, AST, ALT, log_2_GGT, fasting glucose, serum uric acid, TC, TG, LDL-C, HDL-C, smoking status, ex-smoker, exercise status, alcohol consumption, ABI and fatty liver

### The results of subgroup analyses

As is shown in Table 4, the tests of interactions were significant for age and hypertension. (P for interaction = 0.018, 0.022 respectively), while the tests of interaction were not statistically significant for other covariants (*P* values for interaction were larger than 0.05). For younger participants (<60y), an unit increase of eGFR was associated with 2.83 decreased baPWV (− 2.83 (− 4.00, − 1.65)). For elderly participants (> 60 y), the baPWV change was − 6.12 (− 8.64, − 3.60) with every 1 unit increase of eGFR. The change in the elderly population is more pronounced (P for interaction = 0.018). The same trend was also seen in hypertensive people (− 4.55 with hypertension vs − 0.82 with non-hypertension). It was noted that Takuya Fukuda et al. collected the menopausal status in raw data. Therefore, we also adjusted it in the female participants. Compared with no-adjusted menopausal status (− 1.66 (− 3.46, 0.14)), however, the association of eGFR on baPWV (− 1.52 (− 3.26, 0.21)) was not altered after adjusting for menopausal status.

**Table 4:** Results of subgroup analysis and interaction analysis

Characteristic	HR (95%CI)	P for interaction
Sex		0.459
male	−0.80 (− 2.26, 0.66)	
female	−1.66 (− 3.46, 0.14)	
Alcohol consumption		0.518
0–40 g/week	−0.94 (− 2.32, 0.45)	
40–140 g/week	−1.80 (− 4.70, 1.11)	
140–280 g/week	2.08 (− 2.43, 6.59)	
> 280 g/week	− 1.15 (− 5.54, 3.23)	
Regular exercise		0.339
no	−1.39 (− 2.66, − 0.12)	
yes	−0.08 (− 2.56, 2.41)	
Fatty liver		0.178
no	−0.72 (− 2.02, 0.58)	
yes	−2.22 (− 4.18, − 0.27)	
Smoking status		0.627
none	−1.01 (−2.29, 0.27)	
current	−1.64 (−3.96, 0.69)	
Ex-smoker		0.881
no	−0.91 (−2.49, 0.66)	
yes	−1.08 (−2.72, 0.55)	
BMI		0.382
< 18.5	−2.30 (−6.60, 2.00)	
> = 18.5, < 23	−0.63 (−2.12, 0.87)	
> = 23	−1.94 (−3.52, −0.36)	
Hypertension		0.022 #
no	−0.82 (−1.99, 0.35)	
yes	−4.55 (−7.58, −1.53)	
Age		0.018 #
≤60 year	−2.83 (−4.00, −1.65)	
> 60 year	−6.12 (−8.64, −3.60)	
Hyperuricemia		0.172
no	−1.80 (−2.99, −0.62)	
yes	0.53 (−2.68, 3.73)	
FBS status		0.445
≤126 mg/dl	−1.11 (−2.25, 0.03)	
> 126 mg/dl	−5.57 (− 11.46, 0.33)	
TC (tertile)		0.783
Low (117–192 mg/dL)	−1.48 (−3.17, 0.21)	
middle (193–222 mg/dL)	−0.80 (−2.76, 1.17)	
high (223–341 mg/dL)	−1.39 (− 3.36, 0.58)	
TG (tertile)		0.457
Low (12–61 mg/dL)	−0.97 (−2.90, 0.95)	
middle (62–104 mg/dL)	−1.03 (−2.94, 0.88	
high (105–1125 mg/dL)	−1.80 (− 3.93, 0.33)	
HDL-C (tertile)		0.637
Low (23.7–45.6 mg/dL)	−1.93 (−4.03, 0.17)	
middle (45.8–58.2 mg/dL)	−0.58 (−2.50, 1.33)	
high (58.3–108.5 mg/dL)	−1.16 (−2.96, 0.63)	
LDL-C (tertile)		0.623
Low (44–114.4 mg/dL)	−0.80 (−2.43, 0.83)	
middle (115–139 mg/dL)	−1.19 (−3.12, 0.73)	
high (140–233 mg/dL)	−1.94 (−3.77, − 0.11)	

Note1:Above model adjusted for age, sex, BMI, SBp, DBp, AST, ALT, log2GGT, fasting glucose, serum uric acid, TC, TG, LDL-C, HDL-C, smoking status, ex-smoker, exercise status, alcohol consumption, ABI and fatty liver

Note 2**:**In each case, the model is not adjusted for the stratification variable

## Discussion

In the present study, we found the association between eGFR and baPWV had a segmental and different population-specific trend. On the left side of the inflection point (eGFR≤77.05 mL/min/1.73 m^2^), the baPWV was reduced by 2.80 for each additional unit of eGFR. On the right side of the inflection point (eGFR> 77.05), the relationship cannot be observed (1.84 (− 0.50, 4.17), *P* = 0.123). Besides, the stronger association between eGFR and baPWV was detected in elderly and hypertensive populations by subgroup analysis.

A series of studies reported that there was a linear association for renal impairment and arterial stiffness [21–25]. However, most of them did not address nonlinearity, and did not perform the subgroup analysis. Even though Magdalena Madero [26] discovered a nonlinear relationship between arterial stiffness and CKD, they did not interpret this further. Therefore, the contribution of this study was the discovery of a saturating effect on the linear relationship between arterial stiffness and eGFR. In this study, the inflection point we calculated by the recursive algorithm was 77.9 (about 80). The result means the negatively linear association between arterial stiffness and eGFR is only present in participants with relatively abnormal renal function (normal range of eGFR was 90–120). For those with relatively normal renal function, this linear relationship cannot be found. Hirofumi Tomiyama [27] reported that moderate-to-severe impairment of the eGFR was associated with an increase in the arterial stiffness, this is consistent with our findings. In a cross-sectional study including 647 participants, Nakagawa N [28] reported the newly proposed eGFR is significantly associated with arterial stiffness, independent of traditional risk factors for cardiovascular disease. In that study, Nakagawa N did the sensitivity analysis as well. However, the trend of baPWV in different CKD stages was still non-equidistant (CKD1 to 5, baPWVs were 1420 ± 224, 1706 ± 335, 1831 ± 362, 2109 ± 449, 2061 ± 259, 2398 ± 244).

Subgroup analyses are important. These analyses will help us to better understand the independent association of eGFR on baPWV from known risk factors for arteriosclerosis. In the present study, we used risk factors for arteriosclerosis as stratification variables, including age, sex, smoking status, exercise status, fatty liver, BMI, hypertension, uric acid and alcoholic consumption, TC, TG, HDL-C, LDL-C. Effect modifications in age and hypertension were just found. It has been previously reported that eGFR is negatively correlated with baPWV in hypertensive individuals [29–32]. The result is the same as ours. In a review by Marco Matteo Ciccone et al. [33], age has a great influence on arterial stiffness and PWV is a more sensitive index of stiffening in the elderly. In the present study, a stronger association between eGFR and baPWV is detected in older people compared with participants younger than 60 years old. Our findings are consistent with those reported by Matteo Ciccone et al. The role of gender should be considered in depth because it is closely related to arterial stiffness. However, the impact of gender on PWV remains controversial. A large-scale and multi-center survey found that PWV was strongly affected by age and BP, whereas sexual differences in aortic PWV were negligible, even after adjusting for possible confounders [34]. In the present study, the difference of association between eGFR and baPWV is not detected between genders. This finding is identical to previous research.

Our research has the following strengths. Firstly, we performed a sensitivity analysis, which revealed the possibility of a nonlinear relationship. Secondly, we used a generalized additive model (GAM) to clarify the nonlinear relationship. Thirdly, a strict statistical adjustment was used to minimize residual confounding. Fourthly, we calculated the inflection point by recursive algorithm and discovered the saturation effect of eGFR and baPWV by two-piecewise linear regression. Finally, we found that the relationship between eGFR and arterial stiffness was significantly different at different ages and hypertension status, and was consistent with previous studies. In some similar studies published earlier, few of them conducted subgroup analysis.

There are some limitations in our study. Firstly, due to the nature of cross-sectional study, we provide only weak evidence between eGFR and baPWV, and it is difficult to distinguish the cause and effect. Secondly, the research population is limited to the Japanese, so the generalizability is geographically restricted. Thirdly, this study is based on a secondary analysis of published data, so variables that are not included in the data set cannot be adjusted, such as history of atherosclerosis-related diseases.

## Conclusion

The relationship between eGFR and baPWV is non-linear. eGFR is negatively related with baPWV when eGFR is less than 77.05.

## Abbreviations

ABI: ankle-brachial index
baPWV: Brachial-ankle pulse wave velocity
eGFR: Estimated glomerular filtration rate
LDL: Low density lipoprotein
TC: Total cholesterol
